# Paragastric Autonomic Neural Blockade to Prevent Early Visceral Pain and Associated Symptoms After Laparoscopic Sleeve Gastrectomy: a Randomized Clinical Trial

**DOI:** 10.1007/s11695-022-06257-9

**Published:** 2022-09-02

**Authors:** Jorge Daes, David J. Morrell, Andrés Hanssen, Melissa Caballero, Elika Luque, Rafael Pantoja, Jorge Luquetta, Eric M. Pauli

**Affiliations:** 1 Division of Minimally Invasive and Bariatric Surgery, Department of Surgery, Clínicas Portoazul E Iberoamérica, Carrera 30 Corredor Universitario # 1-850, 081007 Puerto Colombia, Colombia; 2 Division of Minimally Invasive and Bariatric Surgery, Department of Surgery, Clínicas Portoazul E Iberoamérica, Carrera 50 no 79-223 PH B, 850020 Barranquilla, Colombia; 3 Division of Minimally Invasive and Bariatric Surgery, Department of Surgery, Penn State Health Milton S. Hershey Medical Center, 500 University Drive, PA 17033 Hershey, USA; 4 Divisiones de Anestesia Y de Cirugía, Universidad Simón Bolívar, Carrera 59 # 59-65, 080020 Barranquilla, Colombia; 5 Department of Anesthesia, Clínicas Portoazul E Iberoamérica, Carrera 30 Corredor Universitario #1-850, 081007 Puerto Colombia, Colombia

**Keywords:** Paragastric, Autonomic, Block, Visceral pain, Opioids, Multimodal pain

## Abstract

**Background:**

Visceral pain (VP) following laparoscopic sleeve gastrectomy remains a substantial problem. VP is associated with autonomic symptoms, especially nausea and vomiting, and is unresponsive to traditional pain management algorithms aimed at alleviating somatic (incisional) pain. The present study was performed to evaluate the safety and effectiveness of laparoscopic paragastric autonomic neural blockade (PG-ANB) in managing the symptoms associated with VP following sleeve gastrectomy.

**Study Design:**

This prospective, double-blinded, randomized clinical trial involved patients undergoing laparoscopic sleeve gastrectomy at two high-volume institutions. The patients were randomized to laparoscopic transversus abdominis plane block with or without PG-ANB. The primary outcome was patient-reported pain scores assessed at 1, 8, and 24 h postoperatively. The secondary outcome measures were analgesic requirements, nausea, vomiting, hiccups, and hemodynamic changes immediately after PG-ANB and postoperatively.

**Results:**

In total, 145 patients (block group, *n* = 72; control group, *n* = 73) were included in the study. The heart rate and mean arterial pressure significantly decreased 10 min after PG-ANB. The visual analog scale score for pain was significantly lower in the PG-ANB than in the control group at 1 h postoperatively (*p* < 0.001) and 8 h postoperatively (*p* < 0.001). Vomiting, nausea, sialorrhea, and hiccups were significantly less prevalent in the PG-ANB group. Patients in the PG-ANB group received fewer cumulative doses of analgesics at 1 h postoperatively (*p* = 0.003) and 8 h postoperatively (*p* < 0.001). No differences between the groups were detected at 24 h (*p* = 0.298). No complications related to PG-ANB occurred.

**Conclusion:**

PG-ANB safely and effectively reduces early VP, associated autonomic symptoms, and analgesic requirements after laparoscopic sleeve gastrectomy.

**Graphical abstract:**

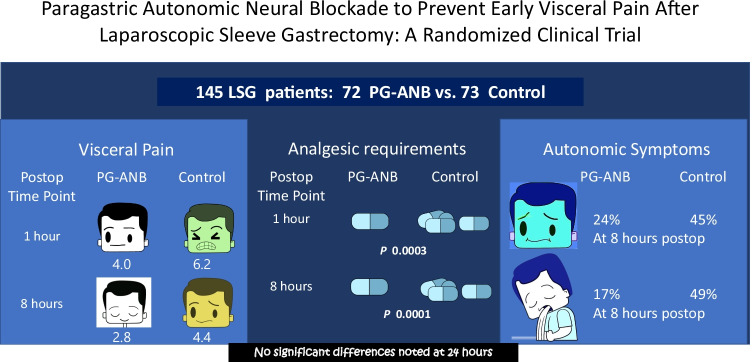

## Introduction

Although seldom discussed, visceral pain (VP) is the most significant source of pain in the first 24 h following laparoscopic sleeve gastrectomy (LSG) and other laparoscopic procedures [1]. The somatic pain induced by surgical trauma to the abdominal wall after LSG is effectively managed using conventional analgesia and transversus abdominis plane (TAP) blocks [2–4]. In contrast, the visceral, colicky pain patients experience after LSG does not respond well to traditional pain management regimens. Patients commonly report VP as epigastric and retrosternal pain that begins immediately after LSG, peaking at 24 h and lasting up to 72 h after LSG [1, 5]. This VP has been ascribed to spasm of the neogastric sleeve, among other factors. VP is often severe and is commonly managed with opioid derivatives [5, 6]. Patients with VP after LSG also have associated autonomic symptoms such as nausea, retching, and vomiting in more than 65% of patients in various reports [7, 8], constituting the most common reason for readmission within 30 days post-surgery [7]. During the last 15 years at our institutions, we have used many pharmacologic strategies to manage these burdensome symptoms in more than 2000 LSG procedures [9, 10]. Despite these efforts, postoperative visceral pain remains challenging to control, often requiring opioids, and postoperative nausea and vomiting (PONV) remain the leading cause of prolonged length of stay and readmissions [5, 7].

Pathways for visceral sensation are diffusely organized both peripherally and centrally. Although the stomach possesses intrinsic neural plexuses that allow some degree of autonomy, it largely depends on extrinsic neural inputs, particularly parasympathetic and sympathetic pathways. The sympathetic nervous system exerts a predominantly inhibitory influence over gastric musculature and motility, with afferent pathways stationed at the celiac ganglion. In contrast, the parasympathetic nervous system, consisting of the vagus nerve and its branches, exerts a predominantly excitatory influence over gastric tone and motility [11]. The anterior and posterior vagus nerves run alongside the lesser curvature of the stomach and branch distally. Sympathetic nerves usually accompany the blood vessels.

Autonomic nerve blocks have been described in the pain management literature. Specifically, celiac ganglia blocks have been reported in managing chronic pain caused by foregut malignancies or pancreatitis [12, 13]. In these patients, neuraxial blocks have been demonstrated to be safe and effective methods of chronic pain management. To our knowledge, however, paragastric autonomic neural blockade (PG-ANB) has not been performed as part of perioperative multimodal pain management algorithms in gastrointestinal surgery. The two proposed main mechanisms of action of this autonomic blockade are a reduction in the parasympathetic influence over the stomach, which reverses its increased muscular tone and deactivates mechanosensitive receptors in the organ wall, and blockade of the afferent sympathetic fibers that convey VP to the spinal cord [14].

In a pilot observational study involving 35 patients, we observed improvement in the severity of VP in the epigastric and retrosternal areas and associated autonomic symptoms following PG-ANB [15]. The effect was most pronounced during the immediate postoperative period but persisted until discharge. Analgesic requirements and the presence of nausea and vomiting were also reduced. The current study was performed to further validate these preliminary findings through a randomized, double-blinded controlled trial.

### Objective

The purpose of this study was to evaluate the safety and effectiveness of PG-ANB in the management of VP in the early postoperative period after LSG. We hypothesized that PG-ANB is safe, reduces VP and associated autonomic symptoms, and provides hemodynamic stability in the early postoperative period after LSG.

## Methods

### Study design

This prospective, double-blinded, randomized clinical trial involved patients undergoing LSG at two high-volume bariatric surgery institutions. The patients were randomized to two parallel groups: LSG with laparoscopic TAP block only and LSG with laparoscopic TAP block and PG-ANB performed as a paragastric lesser omentum neural blockade. The study protocol adhered to the Helsinki Declaration and was approved by the Institutional Review Boards and the Ethics Committee of Clinica Portoazul and Clinica Iberoamerica. The study protocol was registered at ClinicalTrials.gov under the identifier NCT05353426 and published [15]. Furthermore, the reporting of this study complies with the CONSORT 2010 guidelines for reporting randomized clinical trials and includes a flow diagram of progress through the phases of the present parallel randomized trial of two groups: enrollment, intervention allocation, follow-up, and data analysis.

### Patients

Adult patients scheduled for LSG at each participating institution from Aug 25, 2021, to Feb 8, 2022, who consented to study participation were included. The exclusion criteria were an age of < 18 years, the performance of concomitant procedures in addition to LSG, allergies to medications included in the postoperative management protocol, and anesthetic or surgical complications related to the LSG that would alter the postoperative management protocol.

### Study Outcomes

The primary outcome was patient-reported pain scores using an 11-point visual analog scale for pain. The secondary outcomes were analgesic requirements; the presence of nausea, vomiting, hiccups, tachycardia, or hypertension; and the changes in the mean arterial blood pressure and median heart rate 10 min after the blockade. The mean arterial blood pressure and median heart rate in the control group were measured immediately after completing the methylene blue test and 10 min later, corresponding to the exact timing when the measurements were performed in the blockade group. Outcomes were assessed at 1, 8, and 24 h after surgery during the period of inpatient admission following LSG.

### Sample Size

Previously published data have indicated that differences of 1 to 2 points on an 11-point visual analog pain scale are clinically significant [16–18]. Based on these prior studies, we chose a difference of 1 point as the minimum clinically relevant difference for sample size calculation and assumed a standard deviation of 2 points. With a *p*-value of 0.05 and a power of 0.80, we estimated that a total of 128 patients would need to be enrolled in this study. To allow for any potential loss to follow-up, we enrolled 150 patients in the study.

### Randomization and Blinding

Randomization was performed using sealed envelopes prepared by the data manager and stratified by institution in blocks of six. The data manager stored the randomization list containing the final treatment assignments. Only the data manager had access to the randomization list throughout the study. These sealed envelopes were placed in the patient’s charts and could not be opened until the patient was in the operating room under general anesthesia. At that point, the surgeon became cognizant of group assignment but was not involved in assessing study outcomes or collecting data. Both the patient and the investigator assessing and recording study outcomes were blinded to the treatment arm assignments.

### Data Collection

The patient’s age, sex, body mass index, current medications, and medical and surgical history were prospectively recorded at the preoperative clinic visit during study enrollment with informed consent. An analog pain scale survey was administered by an investigator blinded to the patients’ groups at 1 h postoperatively (in the recovery room), 8 h postoperatively, and the following morning. The investigator recorded the need for analgesics; the presence of nausea, vomiting, retching, excessive salivation, and hiccups; and vital signs.

### Statistical Analysis

Continuous outcome variables were compared with two-sample t-tests. Categorical and binary outcome variables were compared using chi-squared tests. Patient-reported pain scores were further compared using linear regression with the surgeon who operated and the location of the procedure as covariates to assess the effect of the surgeon and place on the primary outcome. Given the multiple secondary outcomes (8 secondary outcomes total) assessed at each time point in addition to the primary outcome, Bonferroni adjustment was used to limit type I error in the current study with statistical significance defined as *p*-values < 0.006.

### Anesthesia Protocol

Our two anesthesiologists used the same standard anesthetic protocol: premedication with Lyrica, induction with remifentanil, propofol, and rocuronium, and maintenance with desflurane and remifentanil. The analgesic protocol used in all patients before extubation consists of acetaminophen (1 g) and morphine (3–5 mg). Ondansetron and alizapride were used routinely during induction of anesthesia as antiemetics.

### Surgical Procedures

#### LSG Technique

The LSG technique performed by our group has been previously described in detail elsewhere [9, 10, 15]. Three surgeons from our group (J.D., E.L., and A.H.) with extensive experience in LSG performed all procedures.

#### Laparoscopic TAP Block

The laparoscopic TAP block was performed after testing the sleeve with methylene blue. We infiltrated 40 mL of 50% diluted 0.5% bupivacaine into the posterolateral subcostal area on both sides. We confirmed infiltration into the proper plane by observing the dissemination of the fluid between the transversus abdominis and internal oblique muscle layers. We no longer used sonography to confirm TAP blocks.

#### Paragastric Lesser Omentum Neural Block

**Fig. 1:**
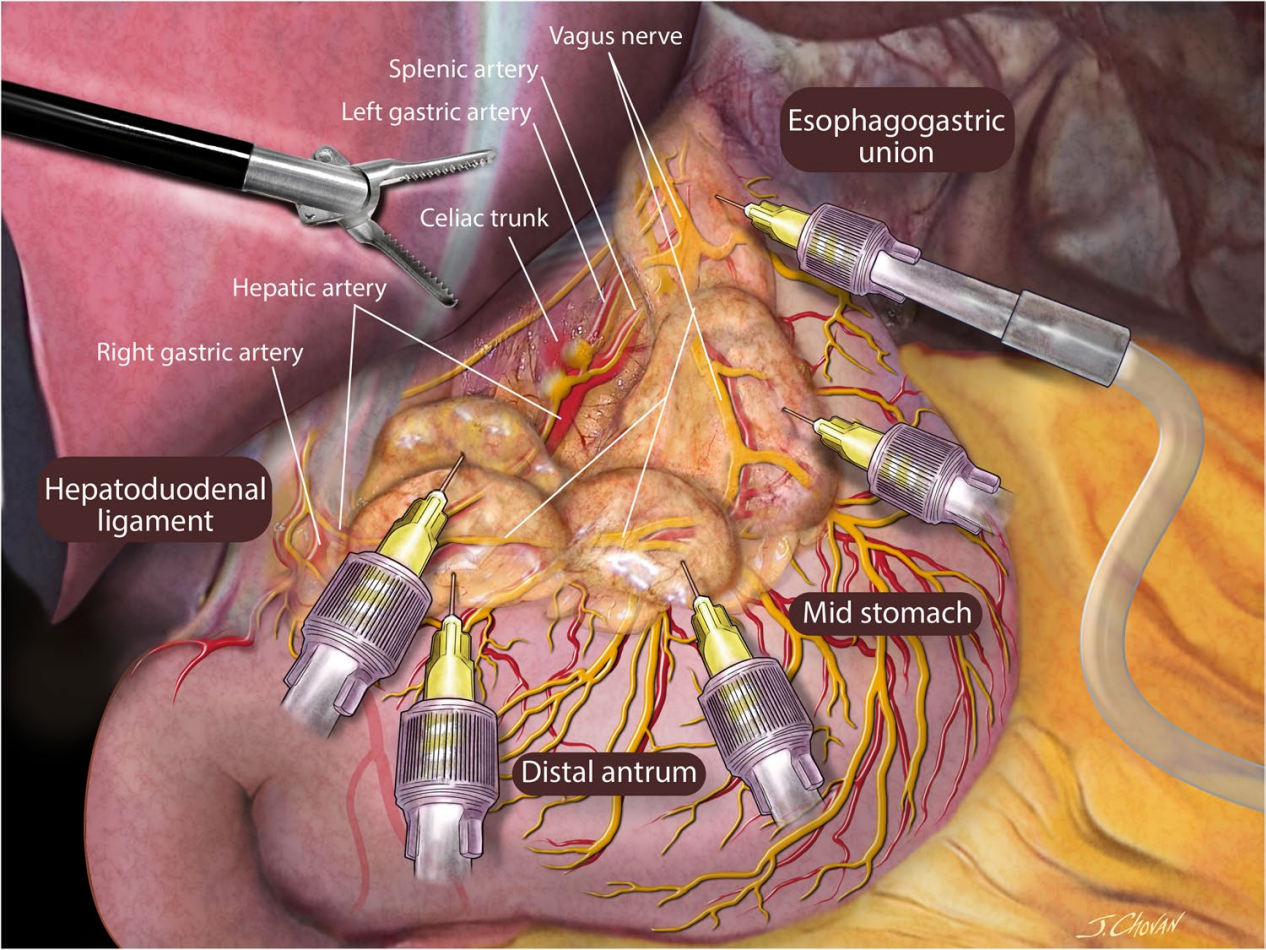
Infiltration was performed at six levels in the fatty tissue of the paragastric area: along the vagus nerve at the esophagogastric junction, at the proximal stomach, at the mid-stomach at the distal antrum, and in the area overlying the hepatic artery

**Fig. 2:**
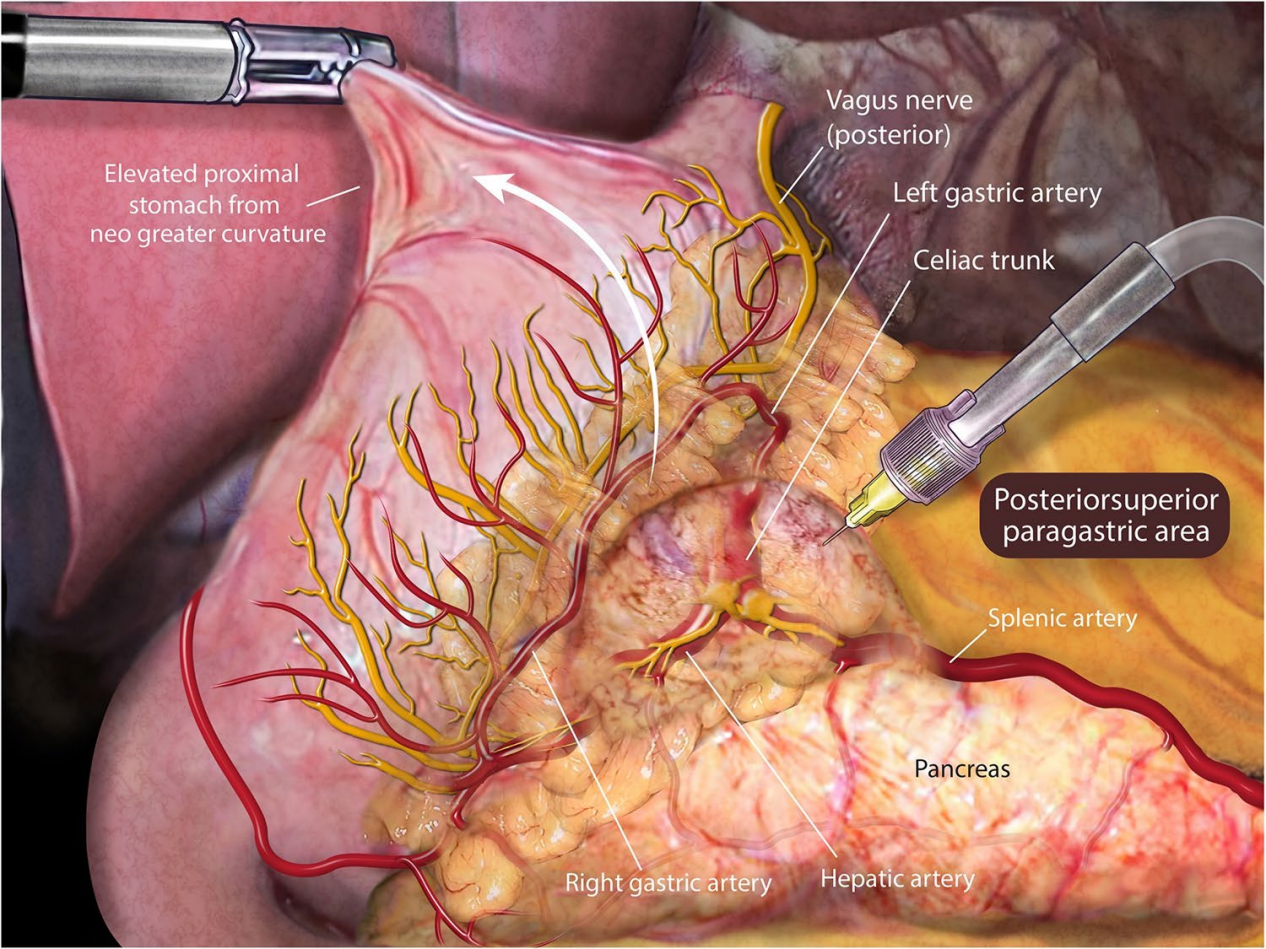
The area overlying the left gastric artery (close to the celiac ganglia) was infiltrated in the posterosuperior paragastric area by elevating the proximal half of the sleeve from the stomach’s neo-greater curvature

The paragastric lesser omentum neural block was performed with a 25-gauge short needle attached to a venous catheter extension introduced through the left 12-mm port. The needle was capped during its introduction, and the cap was removed inside the abdomen using a grasper and kept under direct vision at all times. Infiltration of 20 mL of non-diluted 0.5% bupivacaine was performed at six levels in the fatty tissue of the paragastric area with careful aspiration preceding the infiltration of fluid. We ensured proper infiltration of the lesser omentum along the vagus nerve and distal branches at the esophagogastric junction, proximal stomach, mid-stomach, and distal antrum. Next, we infiltrated the area overlying the hepatic artery (Fig. 1). Finally, the area overlying the left gastric artery was infiltrated in the posterosuperior paragastric area by elevating the proximal half of the sleeve from the stomach’s neo-greater curvature (Fig. 2). The cap was then reapplied to the needle, and the assembly was removed from the abdominal cavity. Details of the procedure are available at https://youtu.be/BGs_1VpuVUw.

### Postoperative Recovery Protocol

All patients received proton pump inhibitors, conventional antiemetics, and a scheduled baseline analgesic such as acetaminophen (1 g intravenously every 6 h) or dipyrone (1 g intravenously every 6 h). The first-line rescue analgesic was a nonsteroidal anti-inflammatory drug such as diclofenac (intravenously every 12 h) together with hyoscine butylbromide (0.2 mg intravenously every 12 h), and the second-line rescue analgesic was Tramadol (1 mg per Kg of ideal body weight intravenously every 6 h). Tramadol was the only opioid derivative used. After surgery, a popsicle was offered in the afternoon, and clear fluids were started the following day. Patients are discharged from the hospital in the afternoon the next day after surgery if they are hemodynamically within normal limits, tolerating clear fluids, and pain is controlled.

## Results

**Fig. 3:**
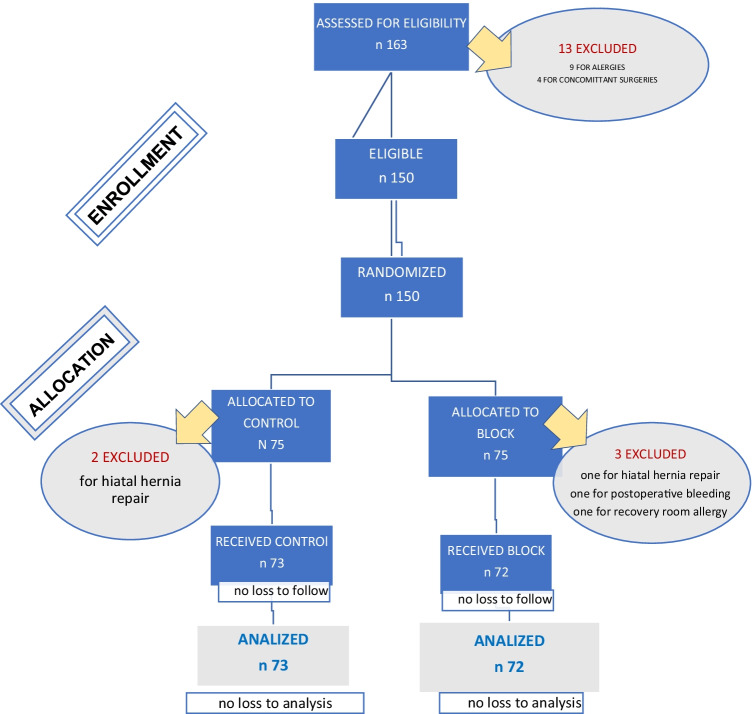
Flow diagram of progress through the phases of the present parallel randomized trial of two groups: enrollment, intervention allocation, follow-up, and data analysis. PG-ANB, paragastric autonomic neural blockade

In total, 163 patients were recruited for study inclusion. Thirteen patients were excluded prior to randomization (nine because of allergies to medications used in the study protocol and four because of a planned concomitant procedure). After excluding these patients, 150 patients were randomized to the PG-ANB group or the control group. As detailed in Fig. 3, five patients were excluded after randomization: three from the PG-ANB group (concomitant hiatal hernia repair, postoperative allergic reaction to a medication requiring deviation from the analgesic recovery protocol, and postoperative bleeding requiring transfusion) and two from the control group (concomitant hiatal hernia repair in both).

Exclusion after randomization was not associated with the study arm assignment (4.0% of PG-ANB cohort excluded, 2.7% of control cohort excluded; *p* = 0.6492). After exclusion, 145 patients were included in the final analysis: 73 in the control group and 72 in the PG-ANB group (Fig. 3). The two groups were comparable in age, sex, body mass index, comorbidities, history of abdominal surgery, and chronic analgesic use (Table 1).

**Table 1:** Baseline patient characteristics

Variable	Block (*n* = 72)	Control (*n* = 73)
Age, *years, mean (SD)*	34.1 (11.0)	35.3 (10.6)
Sex		
Male	27 (37.5%)	22 (30.1%)
Female	45 (62.5%)	51 (69.9%)
BMI, *kg/m*^*2*^*, mean (SD)*	37.7 (4.3)	37.2 (5.8)
Comorbidities		
Hypertension	18 (25.0%)	15 (20.6%)
Diabetes	2 (2.8%)	1 (1.4%)
Insulin resistance	4 (5.6%)	4 (5.5%)
Hyperlipidemia	2 (2.8%)	3 (4.1%)
Obstructive sleep apnea	6 (8.3%)	5 (6.9%)
Gastroesophageal reflux disease	5 (6.9%)	2 (2.7%)
Osteoarthritis	3 (4.2%)	2 (2.7%)
Hepatic steatosis	1 (1.4%)	0 (0%)
Asthma	4 (5.6%)	2 (2.7%)
Depression	1 (2.7%)	2 (1.4%)
History of prior abdominal surgery	36 (50.0%)	34 (46.6%)
Chronic analgesic use	3 (4.2%)	2 (2.7%)

We first evaluated the intraoperative hemodynamic changes that showed a significant difference before and 10 min after the blockade. The mean decrease in heart rate was 9.6 ± 9.6 beats/min in the PG-ANB group and 2.7 ± 11.3 beats/min in the control group (*p* = 0.0001). The mean decrease in the mean arterial pressure was 10.3 ± 9.7 mmHg in the PG-ANB group and 3.3 ± 11.6 mmHg in the control group (*p* = 0.0001) (Table 2). No difference in the heart rate or mean arterial pressure was noted between the two groups at any postoperative time point.

**Table 2:** Perioperative hemodynamic measurements

Variable	Block (*n* = 72)	Control (*n* = 73)	*p*-value
*Intraoperative hemodynamics*			
Heart rate, *bpm, mean (SD)*			
Prior to block	73.7 (11.8)	71.7 (13.0)	0.3185
10 min after	64.2 (9.9)	68.9 (12.5)	0.0115
Difference	− 9.6 (9.6)	− 2.7 (11.3)	0.0001
Mean arterial pressure, *mmHg, mean (SD)*			
Prior to block	73.1 (14.8)	72.3 (12.6)	0.7235
10 min after	62.8 (10.8)	69.0 (11.3)	0.0010
Difference	− 10.3 (− 9.7)	− 3.3 (11.6)	0.0001
*Postoperative hemodynamics*			
Heart rate, *bpm, mean (SD)*			
1 h	78.4 (13.9)	84.3 (14.8)	0.0146
8 h	78.1 (12.3)	79.2 (13.2)	0.5882
Postoperative day 1	75.6 (13.3)	78.1 (13.8)	0.2765
Mean arterial pressure, *mmHg, mean (SD)*			
1 h	101.2 (13.6)	101.8 (18.0)	0.8062
8 h	96.3 (14.1)	98.5 (12.1)	0.3170
Postoperative day 1	98.4 (11.8)	95.6 (11.9)	0.1678

We then evaluated the primary outcome of patient-reported postoperative pain. The visual analog scale score for pain was significantly lower in the PG-ANB group than in the control group at 1 h postoperatively (4.0 ± 2.7 vs. 6.2 ± 2.2, *p* < 0.0001) and 8 h postoperatively (2.8 ± 2.2 vs. 4.4 ± 2.1, *p* < 0.0001). When assessed on the morning of postoperative day 1, no difference was noted between the PG-ANB and control groups (3.1 ± 2.1 vs. 3.0 ± 2.2, respectively; *p* = 0.8174) (Table 3). Notably, in the PG-ANB group, 9 patients denied pain (i.e., visual analog pain scale score of 0) at 1 h postoperatively, and 15 patients denied pain at 8 h postoperatively. In contrast, only two patients in the control group denied pain at 8 h postoperatively. A linear regression analysis was performed to account for potential confounding in patient-reported pain from the effects of the surgeon performing the procedure or the institution where the procedure was performed. After controlling for covariates (e.g., operative surgeon and location of surgery), PG-ANB resulted in a mean decrease in patient-reported pain of 2.3 (95% confidence interval [CI] 1.5 to 3.1) points at 1 h and 1.6 (95% CI 0.8 to 2.3) points at 8 h postoperatively (Tables 4 and 5).

**Table 3:** Postoperative patient-reported visual analog scale scores for pain at 1, 8, and 24 h after laparoscopic sleeve gastrectomy

Time point	Block (*n* = 72)	Control (*n* = 73)	*p*-value
1 h, mean (SD)	4.0 (2.7)	6.2 (2.2)	< 0.0001
8 h, mean (SD)	2.8 (2.2)	4.4 (2.1)	< 0.0001
Postoperative day 1, mean (SD)	3.1 (2.1)	3.0 (2.2)	0.8174

**Table 4:** Linear regression of patient-reported pain at 1 h postoperatively accounting for potential confounding from the effects of the surgeon performing the procedure or the institution where the procedure was performed

Variable	Coefficient	95% CI lower	95% CI upper	*p*-value
Block				
No block	Reference			
Block	− 2.3	− 3.1	− 1.5	< 0.0001
Operative surgeon				
Surgeon 1	Reference			
Surgeon 2	0.6	− 0.3	1.5	0.2034
Surgeon 3	− 1.7	− 3.7	0.2	0.0721
Location of surgery				
Hospital 1	Reference			
Hospital 2	− 0.7	− 1.5	0.1	0.1014
Intercept	6.5	5.8	7.3	< 0.0001
*R*^2^ = 0.2415

**Table 5:** Linear regression of patient-reported pain at 8 h accounting for potential confounding from the effects of the surgeon performing the procedure or the institution where the procedure was performed

Variable	Coefficient	95% CI lower	95% CI upper	*p*-value
Block				
No block	Reference			
Block	− 1.6	− 2.3	− 0.8	< 0.0001
Operative surgeon				
Surgeon 1	Reference			
Surgeon 2	− 0.04	− 0.9	0.8	0.9331
Surgeon 3	− 1.1	− 2.8	0.6	0.2227
Location of surgery				
Hospital 1	Reference			
Hospital 2	− 0.3	− 1.0	0.5	0.4918
Intercept	4.6	3.9	5.2	< 0.0001
*R*^2^ = 0.1376				

Next, postoperative symptoms were evaluated. Nausea and vomiting were less prevalent in the PG-ANB group at 1 and 8 h postoperatively. This was especially evident for vomiting, which was present in 2 and 12 patients at 1 and 8 h postoperatively in the PG-ANB group compared with 20 and 36 patients in the control group (*p* < 0.0001 at both times points) (Table 6).

**Table 6:** Postoperative symptoms at 1, 8, and 24 h after laparoscopic sleeve gastrectomy

Variable		Block (*n* = 72)	Control (*n* = 73)	*p*-value
1 h	Vomiting	2 (2.8%)	20 (27.4%)	< 0.0001
	Nausea	23 (31.9%)	45 (61.6%)	0.0003
	Sialorrhea	19 (26.4%)	35 (48.0%)	0.0073
	Hiccups	0 (0%)	3 (4.1%)	0.0822
8 h	Vomiting	12 (16.7%)	36 (49.3%)	< 0.0001
	Nausea	17 (23.6%)	33 (45.2%)	0.0062
	Sialorrhea	15 (20.8%)	32 (43.8%)	0.0031
	Hiccups	3 (4.2%)	8 (11.0%)	0.1225
Postoperative day 1	Vomiting	35 (48.6%)	35 (49.3%)	0.9324
	Nausea	41 (56.9%)	33 (45.2%)	0.1574
	Sialorrhea	22 (30.6%)	31 (42.5%)	0.1365
	Hiccups	14 (19.4%)	27 (37.0%)	0.0190

**Fig. 4:**
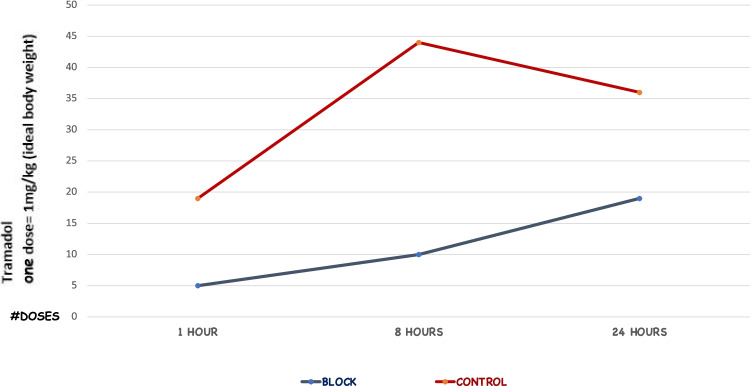
Cumulative opioid doses administered by treatment group (each dose equivalent to 1 mg of tramadol per kg of ideal body weight) administered at 1, 8, and 24 h after laparoscopic sleeve gastrectomy

Finally, we evaluated postoperative analgesic use in the two groups. Patients in the control group received fewer cumulative doses of analgesics at both 1 h postoperatively (*p* = 0.0003) and 8 h postoperatively (*p* = 0.0001) (Table 7). At 24 h postoperatively, there were no differences in the cumulative doses of analgesics between the two groups. When we specifically evaluated the postoperative opioid requirements, we found that the PG-ANB group had used fewer opioid doses at 8 h (*p* = 0.0010) (Table 8). Throughout the postoperative period, roughly three times more total opioid doses were required by the control group than the PG-ANB group at 1 h (14 vs. 5) and 8 h (34 vs. 10) (Fig. 4).

**Table 7:** Cumulative doses of analgesics administered postoperatively at 1, 8, and 24 h after laparoscopic sleeve gastrectomy

Time point	Total doses	Block (*n* = 72)	Control (*n* = 72)	*p*-value
0 to 1 h	0	56 (77.8%)	32 (42.5%)	0.0003
	1	12 (16.7%)	23 (31.5%)	
	2	4 (5.6%)	14 (19.2%)	
	3	0 (0%)	4 (5.5%)	
	4	0 (0%)	1 (1.4%)	
1 to 8 h	0	3 (4.2%)	3 (4.1%)	0.0001
	1	28 (38.9%)	13 (17.8%)	
	2	32 (44.4%)	20 (27.4%)	
	3	8 (11.1%)	29 (39.7%)	
	4	1 (1.4%)	7 (9.6%)	
	5	0 (0%)	1 (1.4%)	
8 to 24 h	0	1 (1.4%)	0 (0%)	0.2977
	1	3 (4.2%)	8 (11.0%)	
	2	28 (38.9%)	16 (21.9%)	
	3	23 (31.9%)	29 (39.7%)	
	4	13 (18.1%)	14 (19.2%)	
	5	3 (4.2%)	4 (5.5%)	
	6	1 (1.4%)	1 (1.4%)	
	7	0 (0%)	1 (1.4%)

**Table 8:** Cumulative doses of opioids administered postoperatively at 1, 8, and 24 h after laparoscopic sleeve gastrectomy

Variable		Block (*n* = 72)	Control (*n* = 73)	*p*-value
1 h	Single dose	5 (6.9%)	10 (13.7%)	0.1394
	Two doses	0 (0%)	2 (2.7%)	
	None	67 (93.1%)	61 (83.6%)	
8 h	Single dose	10 (13.9%)	24 (32.9%)	0.0010
	Two doses	0 (0%)	5 (6.9%)	
	None	62 (86.1%)	44 (60.3%)	
Postoperative day 1	Single dose	13 (18.1%)	11 (15.1%)	0.8878
	Two doses	3 (4.2%)	3 (4.1%)	
	None	56 (77.8%)	59 (80.8%)

No patients developed complications related to the PG-ANB beyond mild, self-limited bleeding in three patients at one of the infiltration sites. All bleeding resolved intraoperatively without further intervention. The hemodynamic changes observed intraoperatively shortly after the block were well tolerated by the patients; in nine patients, the anesthesiologist administered phenylephrine (0.5–1 mg) and atropine (0.5 mg) to regulate the heart rate and mean arterial pressure. One patient developed postoperative bleeding requiring a blood transfusion. This did not require operative reintervention for management and was presumed to have originated from the right paramedian 5-mm port incision, given the extensive ecchymosis in this area. Subsequent sonographic evaluation of the abdomen showed that most of the limited intraperitoneal collection was localized to the right abdomen near this port. There were no leaks or other complications related to the LSG.

## Discussion

Visceral pain is the predominant form of pain after many laparoscopic procedures, in particular LSG. Studies have demonstrated that up to 75% of bariatric patients are discharged from the postoperative recovery room with moderate to severe pain [5]. VP substantially impacts patients’ quality of life, recovery time, nursing time allocation, and resultant risk of opioid abuse [1, 5, 6, 19].

PONV remains a problem in LSG patients, with groups describing a high prevalence (65%) 24 h after surgery despite adequate prophylaxis [7, 20]. PONV increases patient suffering, recovery time, and nursing time allocation and negatively impacts the LOS and the readmission rate after LSG [7]. Ambulatory LSG is gaining popularity, but its rate is still extraordinarily low (3.1%), with readmission rates between 3 to 6.5%, with nausea, vomiting, dehydration, and pain as the most common factors cited for readmission [21]. The level of postoperative symptoms after LSG may also be related to technical aspects such as the size of the bogie used [22]. Many strategies have been described to manage postoperative bariatric symptoms, including intravenous analgesics, magnesium sulfate, opioids, wound infiltration of long-acting local anesthetics, TAP blocks, antiemetics, corticosteroids, and epidural anesthesia (2–4, 6, 23). Prior to this current study, we had implemented an enhanced recovery protocol based on some of these strategies with partial success limiting pain and PONV after LSG.

This randomized controlled trial demonstrated the safety and efficacy of paragastric autonomic blockade after LSG as a multimodal pain and associated symptoms management therapy. The need for analgesics in general and opioids in particular was significantly reduced with the blockade. The control group required 70% and 34% more total doses of analgesics in the first 8 and 24 h after surgery, respectively, than the PG-ANB group. Similarly, the control group required three times more total opioid doses than the PG-ANB group in the same periods. The difference in the incidence of PONV between the PG-ANB and the control group was relevant. The differences in pain scores, associated symptoms, and analgesic requirements disappeared at 24 h postoperatively, paralleling the 0.5% bupivacaine half-life used in PG-ANB. The use of liposomal bupivacaine may improve the blockade duration.

There was a significant reduction in the heart rate and mean arterial pressure 10 min after PG-ANB, which is a possible effect of sympathetic inhibition. We believe this observation early after PG-ANB can be used as an intraoperative marker of an effective blockade. Despite intraoperative differences, heart rate and arterial pressure did not vary between the groups in the postoperative period (at 1, 8, and 24 h).

Based on the literature on predisposing factors to postoperative pain and PONV, there are groups of patients that may especially benefit from the PG-ANB. We believe these patients to include those with allergic reactions to analgesics, chronic opioid users, and patients with chronic pain, previous history of postoperative nausea and vomiting and motion sickness. Ultimately, patient selection for the PG-ANB is beyond the scope of this study which focused on the safety and efficacy of this procedure.

No complications related to PG-ANB occurred besides minimal bleeding in three patients at one of the infiltration sites. All bleeding was resolved intraoperatively by compression with a grasper for 3 min. One patient excluded after randomization developed postoperative bleeding requiring a blood transfusion. This did not require operative reintervention for management. The evidence suggested that the bleeding originated from the right paramedian 5-mm port incision and was not related to the PG-ANB.

The reproducibility of the results of this study is a potential limitation given the introduction of a novel laparoscopic PG-ANB technique. Surgeons who perform these laparoscopic blocks should be skilled minimally invasive surgeons. After a short learning curve, the procedure can be executed relatively fast. The equipment is inexpensive and readily available. Using retractable needles like the Carr-Locke needle may make this procedure more widely adopted. We believe that with the help of the figures and videos in this manuscript, surgeons comfortable with performing LSG and other minimally invasive foregut operations should be able to perform PG-ANB.

Another limitation of the study design is the lack of a placebo control. The decision to omit a placebo control was made after consideration of potential complications from injection during the procedure. Ultimately, the risk from these complications was not felt to outweigh the benefit of a placebo-controlled study design.

This study may represent a new era in the multimodal management of pain and associated symptoms in upper gastrointestinal surgery. With further refinement, targeting pertinent autonomic pathways may allow for expanded indications of other surgical procedures such as colorectal, pancreatic, gallbladder, genitourinary, and other surgeries. The study may lead to several related investigations in the future, including further exploration of strategies to extend the duration of PG-ANB (e.g., use of liposomal bupivacaine, colloids, and adrenaline or creation of blisters of local anesthetics) as well as research on how to refine the timing of the performance of PG-ANB with consideration of performing the block earlier in the procedure (i.e., before the stapled division of the stomach) to potentially diminish anesthetic administration and the hemodynamic changes precipitated by the stomach division (such as tachycardia and hypertension).

## Conclusion

PG-ANB safely and effectively reduces nausea and vomiting, visceral pain, and analgesic requirements in the first 24 h after LSG.

## Abbreviations

VP: Visceral pain
PG-ANB: Paragastric autonomic neural blockade
LSG: Laparoscopic sleeve gastrectomy
TAP: Transversus abdominis plane block
PONV: Postoperative nausea and vomiting
